# Milk-derived exosomes (MDEs) have a different biological effect on normal fetal colon epithelial cells compared to colon tumor cells in a miRNA-dependent manner

**DOI:** 10.1186/s12967-019-2072-3

**Published:** 2019-09-30

**Authors:** Shimon Reif, Yaffa Elbaum Shiff, Regina Golan-Gerstl

**Affiliations:** 1 Department of Pediatrics, Hadassah-Hebrew University Medical Center, Jerusalem, Israel; 2 Institute of Biochemistry and Nutrition, The Robert H. Smith Faculty of Agriculture, Food and Environment, The Hebrew University of Jerusalem, Rehovot, Israel

**Keywords:** Milk, Exosomes, miRNA, Colon cells

## Abstract

**Background:**

Breastfeeding is the ideal source of infant nutrition. Human milk consists not only of nutrients but also biologically active components. Among these latter compounds, exosomes contain proteins, lipids, mRNAs and miRNAs.

**Methods:**

To elucidate the biological effects of milk-derived exosomes (MDEs) on normal colonic epithelial cells compared to colonic tumor cells, we incubated cells with MDEs. MDEs were able to enter into normal and tumor cells and change their miRNA expression profiles. Proliferation, cell morphology and protein expression were analyzed in these cells.

**Results:**

Human milk-derived exosomes induced proliferation- and epithelial mesenchymal transformation-related changes, such as collagen type I and *twist* expression, in normal but not in tumor cells. PTEN, a target of miRNA-148a, was downregulated in normal but not in tumor cells following incubation with MDEs. Moreover, miRNA-148a-3p knockdown cells were used to demonstrate the importance of miRNA in the effect of exosomes on cell proliferation and protein expression. MDEs inhibited proliferation and DNMT1 expression in cells with knockdown of miRNA-148a.

**Conclusions:**

In conclusion, the positive effect of exosomes on normal cells without affecting tumor cells may presents an aspect of their safety when considering it use as a nutritional supplement to infant formula.

## Background

Breast feeding is the ideal source for infant nutrition. Human milk is recognized as one of the most valuable contributors to infant health, leading to adequate growth of the brain and the immune system [1]. Previous studies showed that part of the protective effects of breast milk occurred via inhibition of enterocyte apoptosis and restoration of enterocyte proliferation and differentiation [2].

Human milk includes not only nutrients, but also bio active compounds that have an important role in various health benefits. Exosomes in mammalian milk were found by us and others and they include bioactive components. These nano vesicles (30–160 nm) are released from a variety of cells and carry a cargo of proteins, lipids, mRNAs, and miRNAs which can be transferred to diverse locations in the body [3, 4]. Exosomes protect miRNA from degradation and transfer its components such as mRNAs into the intestine by facilitating uptake and endocytosis. Milk derived exosomes (MDE) have also a special significance because they have the capacity of transferring genetic information from mother to infant [5, 6].

There is a previous study that demonstrate that porcine milk exosomes and their miRNAs are taken up by intestinal epithelial cells, modify target gene expression and promote proliferation of intestinal cells There is a previous study that demonstrate that porcine milk exosomes and their miRNAs are taken up by intestinal epithelial cells, modify target gene expression and promote proliferation of intestinal cells There is a previous study that demonstrate that porcine milk exosomes and their miRNAs are taken up by intestinal epithelial cells, modify target gene expression and promote proliferation of intestinal cell MiRNAs are small non‐coding RNAs involved in post‐transcriptional gene regulation. They control a wide range of cellular functions such as cell differentiation, proliferation, and apoptosis [7, 8]. Dysregulated expression of miRNAs may play an important role in the development of diseases such as cancer [9, 10] and autoimmune disease, which are both conditions that related to cell proliferation. We previously showed that exosomes in mammalian milk contain beneficial miRNAs such as miRNA-148a-3p (miRNA-148a) [11]. We showed that MDE can enter into cells, and they can regulate biological and cellular functions such as gene expression. Micro RNA-148a have been shown to have multifaceted function, including control of cellular proliferation and differentiation, as well as apoptosis.

Milk derived exosomes were shown to inhibit cell growth of different cell lines [12, 13]. On the other hand, there are different studies that demonstrate induction of cell proliferation by MDE [14–16]. Based on this different effect of MDE we hypothesize that MDE exosomes have a different regulatory effect on tumor and normal cells. In the current study, our aim was to compare the effect of MDE on colonic fetal normal and tumor cells. We planned to study whether the effect of MDE is via miRNA. To elucidate the effect of MDE on normal epithelial cells compared to tumor cells, we incubated normal epithelial and colonic tumor cells with MDE. Proliferation, morphology and protein expression were analyzed in different cell types. MIRNA-148a knockdown cells were used to demonstrate the importance of miRNA to the effects exerted by exosomes on cell proliferation and protein expression.

## Methods

Ethical approval information: This study was approved by the Investigational Review Board (IRB) of Hadassah-Hebrew University Hospital (HM0-0101-13). All mothers participating in the study signed an informed consent form approved by the IRB.

### Milk sample collection and fractionation

Milk samples were collected from healthy mothers during the first 60 days after delivery. Cow milk was collected before pasteurization from a pool of milk. Samples were transported to the laboratory and stored at − 80 **°**C until further analysis. They were fractionated by centrifugation at 6500*g* for 30 min at 4 °C. Fat and skim milk fractions were obtained from each sample. The skim milk and the lipid layer were transferred separately to different tubes. Skim milk was centrifuged at 12,000*g* for 1 h at 4 °C to remove debris. The defatted supernatant was then passed through 5 μm and 0.45 μm filters to remove residual debris.

### Exosome isolation

Exosomes from human milk were isolated from the skim fraction of the milk following a series of centrifugations and filtrations as described above. Exosome isolation was performed as described previously by us with ExoQuick reagent (System Biosciences, Palo Alto, CA, USA) according to the manufacturer’s instructions [11]. Briefly, 63 μl of ExoQuick was added to 250 μl of the skim milk fraction, and the mixture was incubated overnight at 4 °C with no rotation. Two centrifugation steps were then performed at 1500*g* for 30 and then 5 min to sediment the exosomes, and the pellet was resuspended in 200 μl of phosphate buffered saline (PBS). MDEs from cow milk were isolated as described in the supplementary data.

### Electron microscopy

Exosomes were analyzed by electron microscopy using negative staining. Isolated exosomes were stained with 2% phosphotungstic acid (PTA) in water. Briefly, 5 μl of diluted exosomes in PBS was placed on Formvar/carbon-coated copper 200 mesh grids (EMA) and mixed with 5 μl of PTA for 10–20 s. Excess stain was blotted off, and the grids were dried. Samples were examined with a Jem-1400 *Plus* transmission electron microscope (Jeol, Peabody, MA, USA).

### Dynamic light scattering (DLS)

The size distribution of the MDEs was measured by a Zetasizer instrument (Malvern Nano‐Zetasizer), as described previously for characterizing the MDEs [17, 18]. Diluted MDEs in PBS were analyzed by a monochromatic laser beam with a detection angle at 173°. Measurements were taken at 25 °C. The exosome size data refers to the scattering intensity distribution.

### Cell culture

LS123 colonic cancer cells and CCD 841 normal colon epithelial cells were grown in Eagle’s Minimum Essential Medium (MEM) supplemented with 10% fetal calf serum (FCS), 100 U/ml penicillin, and 100 μg/mL streptomycin in a humidified incubator (37 °C, 5% CO_2_).

293T cells were grown in Dulbeco’s modified Eagle’s medium (DMEM) supplemented with 10% FCS penicillin and streptomycin. To generate stable transductant pools, 293T cells were infected with pGreenPur MiRZip lentivectors (System Biosciences, San Francisco, CA, USA) expressing short hairpin RNA to miRNA-148a and containing the copGFP gene. At 24 h after infection, the medium was replaced, and 24 h later, infected cells were selected with puromycin (2 μg/mL) for 96 h.

### Exosome labeling

RNA in the MDEs was labeled using the Exo-Glow Exosome Labeling Kit (System Biosciences, San Francisco, CA, USA). We added 20 μl of 10× Exo-Red to 200 μl of a resuspended exosome suspension in PBS. The mixture was mixed well by flicking/inversion and incubated for 10 min at 37 °C. To stop the labeling reaction, we added 40 μl of the ExoQuick-TC reagent to the labeled exosome sample suspension and mixed it by inverting six times. The labeled exosome samples were incubated on ice for 30 min. Samples were then centrifuged at 12,000 g for 3 min to sediment the exosomes, and the pellet was resuspended in 200 μl of PBS.

### Extraction of total RNA

MDEs were incubated with the cell lines in starving medium (0% FCS). After 24 h, the cells were washed with PBS and detached from the plate using trypsin. Cells were collected and centrifuged at 1600 rpm for 5 min. Pellets were resuspended with TRIzol reagent (Invitrogen, Paisley, UK) for total RNA isolation as described below. Chloroform was added to the pellet cells; the mixture was then shaken vigorously, incubated for 15 min at room temperature, and centrifuged at 12,000*g* for 15 min at 4 °C. The aqueous phase was carefully transferred to a new tube. Subsequently, isopropanol (0.5 ml per 1 ml of TRIzol reagent) was added to precipitate the RNA, and the solution was mixed by inversion. Following incubation for 10 min at room temperature, the samples were centrifuged at 12,000*g* for 10 min at 4 °C. The supernatant was discarded, and the pellet was washed with 75% ethanol (1 ml per measure of TRIzol reagent) and centrifuged at 12,000*g* for 5 min at 4 °C. The pellet was air dried and resuspended in RNase-free water. RNA quantity and quality were assessed by measuring the absorbance of the RNA samples at different wavelengths using a NanoDrop spectrophotometer. We used RNA with a 260/280 ratio of at least 1.80 in nuclease-free water. A 260/230 ratio was also used to estimate the presence of other contaminants.

### Quantification of mRNA by qRT-PCR

Complementary DNA for the quantification of mRNA was generated using the high capacity RNA-cDNA kit (Applied Biosystems, Foster City, CA, USA) according to the manufacturer’s instructions. Total RNA isolated from cells (1 µg) was used to generate cDNA. The cDNA was subjected to qPCR. The mRNA levels of fatty acid synthase (FAS1) were measured using qRT-PCR with Fast SYBR Green master mix (Applied Biosystems) using a StepOne Plus Real-Time PCR System machine (Applied Biosystems). Primers: *twist* For: 5′-GGAGTCCGCAGTCTTACGAG-3′ Rev: 5′-TCTGGAGGACCTGGTAGAGG-3′, *β*-*actin* For: 5′-AGAAAATCTGGCACCACACC-3′ Rev: 5′-AGAGGCGTACAGGGATAGC-3′, *GAPDH* For: 5′-GATCATCAGCAATGCCTCCT 3′; Rev: 5′ TGTGGTCATGAGTCCTTCCA 3′. The PCR steps were 1 cycle at 95 °C for 5 min, 40 cycles of 95 °C for 5 s, and 60 °C for 30 s. The 2^(-ΔΔCT) method was used to determine the relative amounts of mRNA.

### MicroRNA detection by qRT-PCR

From the total RNA samples, 400 ng of total RNA was used to prepare cDNA using the qScript microRNA cDNA Synthesis Kit (QuantaBio, Beverly, MA, USA). After cDNA synthesis, an equivalent of 2.4 ng of the original RNA sample was mixed with Perfecta SYBR Green SuperMix (QuantaBio) and Universal PCR Primers (QuantaBio) in 15 μl of qPCR reactions. Three cDNA samples were run in adjacent wells of each 96-well qPCR plate. The qPCR plates were run using the StepOnePlus Real-Time PCR System (Applied Biosystems) using a two-step cycling protocol (95 °C for 5 min followed by 40 cycles of 95 °C for 5 s and 60 °C for 30 s), concluding with a melting curve. After the reactions were completed, Ct values were determined using fixed-threshold settings. The 2^(− ΔΔCT) method was used to determine the relative amounts of miRNAs.

### Immunoblotting

Cells were lysed in SDS, separated by SDS-PAGE, and transferred onto a PVDF membrane. The membranes were probed with antibodies and detected using enhanced chemiluminescence detection. Primary antibodies were as follows: anti-β-catenin (1:2000; Sigma Aldrich, St Louis, MO, USA), anti-β-actin (1:1500; R&D Systems, Minneapolis, MN, USA), anti-collagen Iα1 (Affinity Bioreagents, Golden, CO, USA), anti-DNMT1 (1:1000; Cell Signaling Technology, Danvers, MA, USA), anti-PTEN (1:1000; Epitomics, Burlingame, CA, USA), anti HSP70 (1:1000; SBI System Biosciences, Palo Alto, CA, USA), and anti CD81 (1:1000; Cosmo Bio, Tokyo, Japan). The secondary antibody was horseradish peroxidase (HRP)-conjugated goat anti-mouse or anti-rabbit (1:3000; Cell Signaling Technology).

Quantification was performed using NIH-Image software (http://rsb.info.nih.gov/nih-image/download.html).

### Growth curves

Cell growth was determined by a microculture methylene blue assay [19]. Cells were fixed in glutaraldehyde at a final concentration of 0.05% for 10 min at room temperature. After washing, the microplates were stained with 1% methylene blue in 0.1 M borate buffer, pH 8.5, for 60 min at room temperature. The plates were then washed extensively and rigorously to remove excess dye and dried. The dye taken up by cells was eluted in 0.1 N HCl for 60 min at 37 °C, and absorbance was monitored at 620 nm. Each point of the growth curve experiments was calculated from 4 to 5 wells. The cells were photographed after fixation and methylene blue staining.

### MTT [3-(4,5-dimethylthiazol-2-yl)-2,5-diphenyltetrazolium bromide assay

Proliferation was measured by CellTiter96 nonradioactive cell proliferation kit (Promega Corp., Madison, WI) and MTT based assay, according to the manufacturer’s recommendations. Briefly, cells were seeded in a 96-well plate overnight and incubated with MDEs for 24 h. At the end of the treatment period, the medium was changed and incubated with the dye solution for 4 h, at 37 °C followed by solubilization in dimethyl sulfoxide and spectrophotometric measurement. The rate of formazan dye formation was determined by measuring the absorbance (570 nm–640 nm). The 570 nm–640 nm reading value is directly proportional to the number of living cells.

### Statistical analysis

**Fig. 1:**
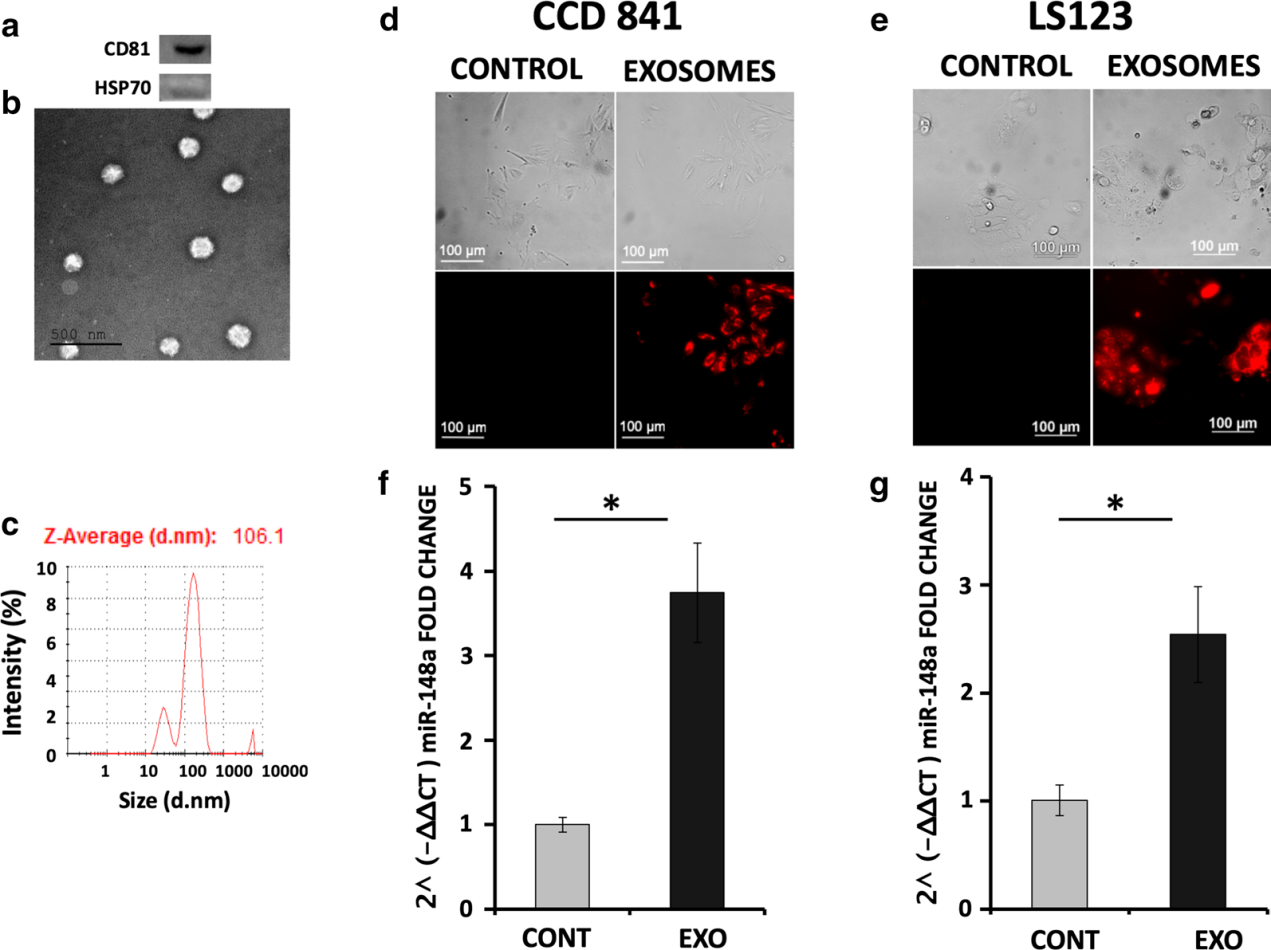
Isolation of milk-derived exosomes (MDEs) and their uptake by different tumor and normal colon cells. Protein expression of CD81 as a marker of exosomes and HSP70 as a negative control in MDEs (**a**). MDEs were analyzed by transmission electron microscopy using negative staining (**b**). Particle size distribution (by intensity) of isolated MDEs determined by DLS (**c**). Labeled milk exosomes isolated from human milk were incubated for 4 h with normal intestinal (CCD 841) or colon cancer (LS123) cells. Images were obtained by fluorescence microscope analysis (**d, e**). MiRNA-148a expression in CCD 841 and LS123 cells incubated with (EXO) or without (CONT) MDEs was analyzed by qRT-PCR (**f, g**). qRT-PCR results were calculated using the 2^(− ∆∆CT) method, and values were normalized against RNU6, *p < 0.05 *t* test

**Fig. 2:**
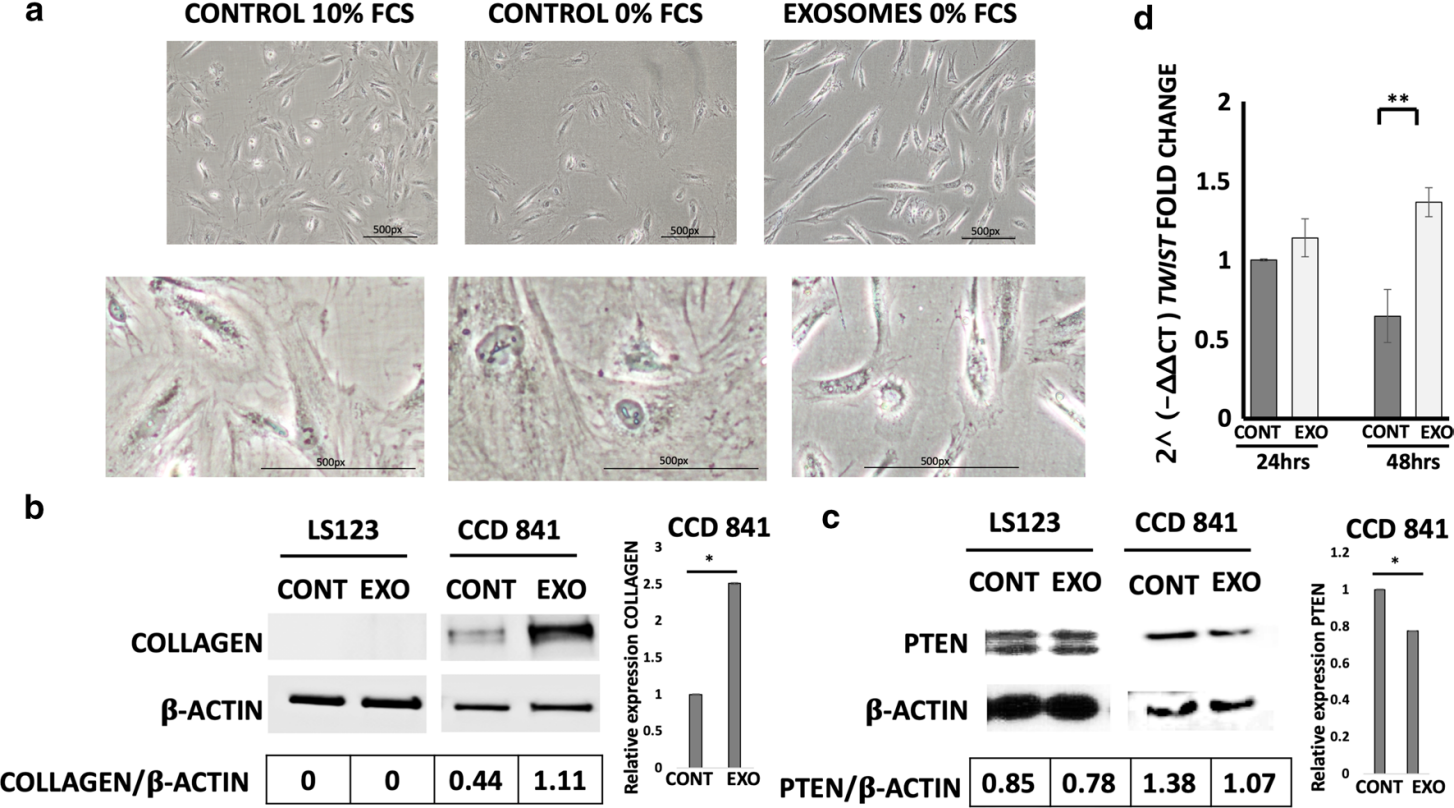
Morphology of normal colon cells incubated with MDEs. MDEs were grown in 10% or 0% FCS and incubated with normal colon cells (CCD 841). Light microscope pictures of selected fields of CCD 841 cells incubated with (EXOSOMES) or without (CONTROL) MDEs (**a**). Expression of collagen-type I (COLLAGEN) and PTEN protein were analyzed in normal colon cells (CCD 841) and colon tumor cells (LS123) by Western blot Quantification was performed with NIH-Image software (http://rsb.info.nih.gov/nih-image/download.html), *p < 0.05 (**b, c**). The expression of the *twist1* gene (*TWIST*) in CCD 841 incubated with MDEs was analyzed by qRT-PCR using the 2^(− ∆∆CT) method, and the values were normalized against β-actin and GAPDH, **p < 0.01 (**d**)

**Fig. 3:**
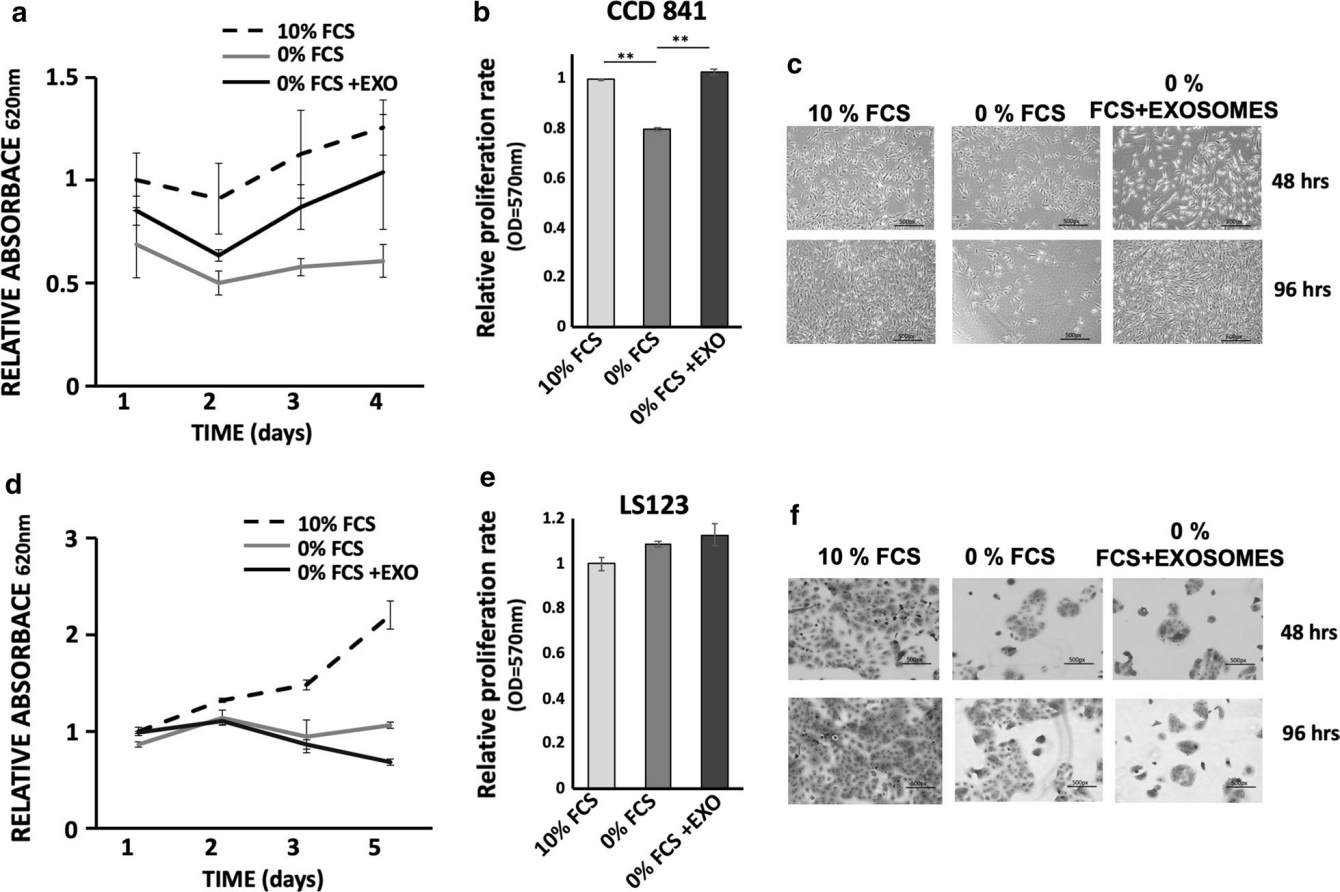
Proliferation of normal colon and colon tumor cells incubated with MDEs. Cell proliferation of CCD 841 and LS123 cells grown in 10% FCS (10% FCS) or 0% FCS incubated with MDEs (0% FCS +EXO) or without (0% FCS) was examined by methylene blue staining (**a**, **d**) and MTT assay (cells were incubated for 24 h with MDEs) (**b**, **e**). Error bars represent SD (n = 5) (**a, d**) SD (n = 4) (**b**, **d**) *p < 0.05, **p < 0.01. Light microscope pictures of selected field cells (**c**, **f**)

**Fig. 4:**
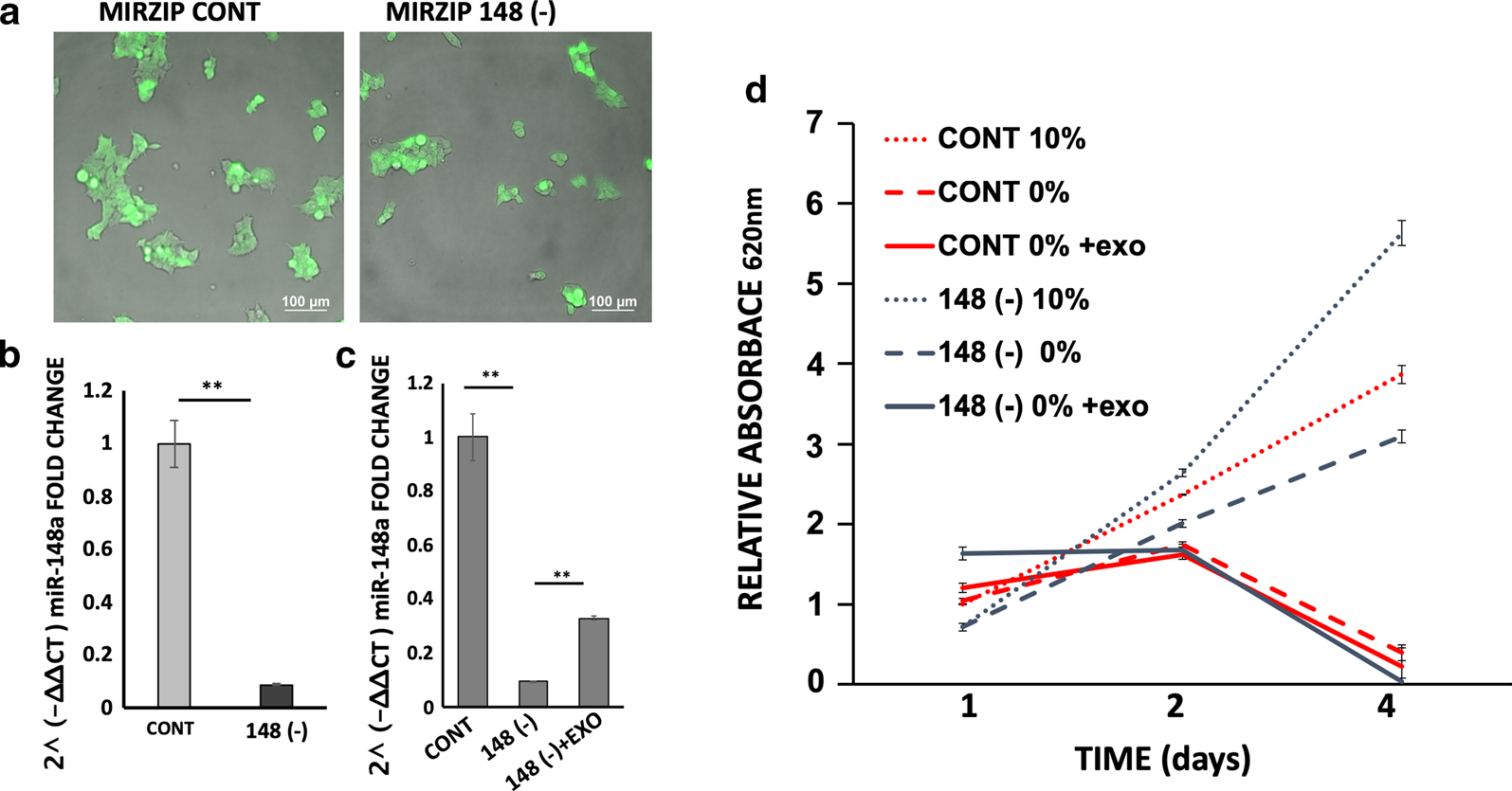
Proliferation of 293T miRNA-148a knockdown cells incubated with MDEs. 293T cells were transduced with lentivirus encoding miRNA-148a-specific shRNA (**148 (−)**) or a scrambled sequence (**CONT**). After selection, cells were analyzed for miRNA148a expression by fluorescence microscopy (**a**) and by qRT-PCR. qRT-PCR results were calculated by the 2^(− ∆∆CT) method, and values were normalized against RNU6B (**b**). MiRNA-148a expression following incubation of miRNA-148 (−) cells with MDEs (**c**). Cell proliferation of cells grown in 10% FCS (10%) or 0% FCS (0%) and incubated with MDEs (MIRZIP 148A 0% +exo) or without (MIRZIP CONT 0% +exo) was examined by methylene blue staining (**d**)

**Fig. 5:**
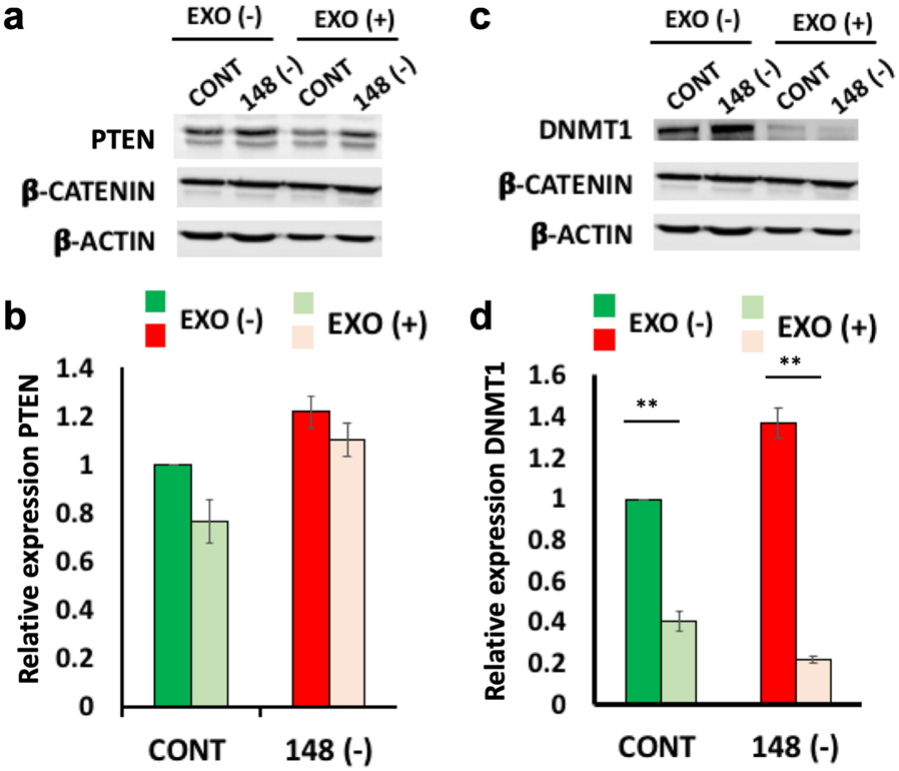
Expression of PTEN and DNMT1 in 293T cells with miRNA-148a knockdown and incubated with MDEs. Expression of PTEN (**a**) and DNMT1 (**c**) proteins was analyzed in 293T miRNA-148a knockdown cells following incubation with MDEs. Protein expression was determined by Western blot. β-Catenin and β-actin were used as loading controls. Quantification was performed with NIH-Image software (http://rsb.info.nih.gov/nih-image/download.html) (**b** and **d**) **p < 0.01

All data were presents as mean ± standard error of mean (SEM). Statistical analysis in experiments with two groups was performed by student-test analysis (Figs. 1f, g, 2a, b and 4b). Differences were considerate to be statistically significant if the p value was < 0.05. For comparison of more than two groups, we used one-way analysis of variance, followed by Tukey’s multiple comparison (Figs. 2d, 3b, 4c and 5d). Values < 0.05 were considered statistically significant. The level of statistical significance is indicated throughout with * for p < 0.05 and ** for p < 0.01.

## Results

### Milk-derived exosomes are able to enter into normal and tumor cells and alter their mRNA expression profiles

To study the ability of MDEs to enter into normal cells compared to tumor cells, the exosomes were incubated with the different types of cells. The isolated vesicles were identifiable as exosomes based on protein composition (Fig. 1a) and electron microcopy examination (Fig. 1b). The Zeta-average (d-nm) of the MDEs was 106.1 nm, as shown in Fig. 1c. Isolated labeled exosomes from breast milk were incubated with CCD 841 cells (human normal fetal colon epithelial cell line) and LS123 (colon tumor cells) (Fig. 1d, e). All the normal and cancer cells were visualized by fluorescence microscopy and were positively stained, which indicates that the exosomes and their RNA content were taken into those cells. Moreover, the expression of miRNA-148a, a highly expressed miRNA in milk exosomes, was upregulated following incubation (Fig. 1f, g).

### Morphological changes of normal epithelial cells induced by milk-derived exosomes

We studied the effect of human and cow MDEs on several biological functions, such as cell morphology and cell growth. MDEs induced morphological changes in normal colonic epithelial cells (Fig. 2a and Additional file 1), which changed from their classic cuboid shape to a mesenchymal-like shape. In contrast, the morphology of the colonic tumor cells was not affected by incubation with exosomes (Fig. 2b and Additional file 2).

We determined the expression of collagen type I in the different cell types. MDEs upregulated the expression of collagen type I in normal epithelial cells but not in colonic tumor cells (Fig. 2b). MDEs also downregulated *twist1* gene expression and phosphatase and tensin homolog (PTEN) protein in normal but not in colonic tumor cells (Fig. 2d, c).

### Milk-derived exosomes contribute to cell proliferation of normal but not tumor cells

To further examine whether MDEs play a role in the proliferation of normal and tumor cells, we measured the proliferation rates of CCD 841 and LS123 cells incubated with milk-derived exosomes. Starvation medium reduced the growth rate of normal and tumor cells compared to the rates in 10% FCS media (Fig. 3a, d). However, incubation of normal cells in starvation conditions but with exosomes increased the proliferation rate (Fig. 3a–c). In contrast, MDEs did not increase tumor cell proliferation under starvation conditions (Fig. 3d–f).

### The effect of tumor cell growth is dependent on miRNA-148a expression

We demonstrated that the miRNA profile expression in cells is changed by incubation with exosomes (Fig. 1). To examine whether miRNA-148a has a major function in cell proliferation, stable miRNA-148a was knocked down in 293T tumor cells. 293T cells that expressed stable shRNA-to-miRNA (148 (−)) and control (**CONT**) cells were GFP positive (Fig. 4a). miRNA 148a expression was downregulated in 148 (-) cells compared to control cells (Fig. 4b).

miRNA-148a silencing in tumor cells upregulated their proliferation (Fig. 4c). Starvation growth conditions decreased proliferation in 293T control cells compared to 293T 148 (−) cells. MDEs reduced the ability of 293T 148 (−) cells to proliferate under starvation growth conditions (Fig. 4).

### PTEN and DNA methyltransferase 1 (DNMT1) expression is downregulated following incubation with milk-derived exosomes

PTEN and DNMT1 are target genes of miRNA-148a that are downregulated by miRNA-148a. The expression of PTEN protein was downregulated in normal cells after incubation with exosomes. In contrast, PTEN expression in tumor cells did not change following incubation with MDEs (Fig. 5). PTEN and DNMT1 expression was shown to be miRNA-148a dependent (Fig. 5) and was downregulated by incubation of 293T control cells (CONT) with exosomes. Inhibition of miRNA-148a in 293T knockdown cells (148 (−)) induced PTEN and DNMT1 expression; however, the upregulation was inhibited following incubation of 293T 148- with exosomes.

## Discussion

Exosomes can be found in most human biological fluids, including blood, urine, saliva and milk. Breast milk, however, is known to contain the highest content of exosomes and is thus considered to be a rich source. Exosomes are nanoparticles containing a wide variety of proteins, lipids, mRNA and miRNA protected by a phospholipid membrane [20, 21]. They provide biological communication during lactation between mothers and their babies and thus enable the transfer of genetic material from mothers to their babies through the milk. We and others have shown that exosomes are bioactive products that transfer miRNAs into cells and induce biologic changes in them [6, 11, 22]. Milk derived exosomes were shown in previous studies to inhibit cell growth of different cell lines [12, 13]. On the other hand, there are other studies that demonstrate induction of cell proliferation by MDE [14–16]. We compared in the same conditions the effect of MDE on proliferation, EMT, gene and protein expression of normal and intestinal cells. We focused on intestinal cells in the present study because the intestine is the first target organ of milk-derived exosomes.

Here, we show that human MDEs enter into normal and tumor epithelial intestinal cells, but with differing effects. Epithelial–mesenchymal transition (EMT) is a type of epithelial plasticity that is characterized by long-lasting changes from an epithelioid to a mesenchymal/fibroblastoid/spindle-shaped morphology [23]. EMT occurs during normal developmental processes, including mesoderm and neural tube formation, as well as in wound healing and cancer progression [24, 25]. We hypothesize that induction of EMT in embryonic intestinal cells can be involved in the development of the intestine. In carcinogenesis, EMT is considered to play an important role in invasiveness and metastasis [26]. For instance, in breast cancer cells, milk exosomes containing high levels of TGFβ2 promote EMT [27]. In contrast, we found that in epithelial colon cells, exosomes have different effects related to EMT changes in normal and tumor cells. Morphological and collagen protein expression changes were induced in normal fetal epithelial cells but not in tumor cells. MDEs induced fetal epithelial cells to change from their classic cuboid shape to a mesenchymal-like shape and upregulated collagen type I expression. EMT can be initiated by overexpression of certain proteins, e.g., Twist family bHLH transcription factor 1 (*twist1*) [28]. Twist is one of the markers of EMT, and it promotes EMT in normal epithelial cells such as HMLE [28]. We indeed showed that exosomes induced the upregulation of *twist* expression on epithelial normal cells (Fig. 2).

MiRNAs in milk are involved in various physiological functions, including the regulation of cell growth, proliferation, and EMT, among others [6, 16, 27]. In our previous study, we found that miRNA 148a is a highly expressed miRNA in milk. In the current study, we showed that miRNA-148a can be transferred to epithelial cells by incubation with MDEs and can regulate their target gene expression [11]. We further showed that the expression of PTEN, a target gene of miRNA-148a, was downregulated in normal cells following incubation with exosomes. Nevertheless, in tumor cells, exosomes only slightly downregulated PTEN expression.

The tumor suppressor gene PTEN is a key regulator in tumorigenesis and tumor development [29]. PTEN inhibits cell proliferation, promotes cell apoptosis and causes cell cycle arrest [30]. During the process of reprogramming mouse embryonic fibroblasts (MEFs), the inhibition of PTEN promotes their proliferation [31].Moreover, the loss of PTEN induces EMT [32]. MDEs may induce proliferation and EMT changes in normal cells by down-regulating PTEN expression via miRNA-148a. In tumor cells, PTEN expression was not significantly affected by MDEs, explaining in part the loss of proliferation induction and EMT-related changes in these cells. Taken together, these findings suggest that miRNA-148a can induce proliferation and EMT changes via PTEN in a cell type-dependent manner. Milk-derived miRNA-148a may induce proliferation and EMT in normal cells but inhibit EMT in tumor cells partly by inhibiting PTEN expression. In contrast, we found that there was more proliferation in 293T 148 (−) cells induced by MDEs in comparison to naïve 293T cells. However, we found higher expression of PTEN in 293T (148-) cells. This may be explained because miRNA-148a regulation of proliferation may be greater than the regulation of PTEN. The effect of MDEs on proliferation may be due to other genes targeted by miRNA-148a.

MDEs are capable of enhancing intestinal tract development in mice by inducing the upregulation of proliferation-related gene expression, such as that of CDX2 and PCNA [6]. Additional research has shown the induction of proliferation of epithelial cells by MDEs isolated from rat [16] and porcine [6] milk. However, no comparison was performed between normal and tumor cells, and no mechanism was proposed. In our study, we compared the effect of human MDEs on normal human colon epithelial cells and colon tumor cells and induced proliferation in normal but tumor cells.

High miRNA-148a expression has been shown in a variety of tissues, including the brain, heart, liver, thymus, pancreas, kidney, placenta, uterus, and hematopoietic system [33, 34]. Highly expressed miRNA-148a was shown to be involved in the regulation of gene expression to maintain gastric tissue stability, and its abnormal expression may induce gastric neoplasms [35]. Likewise, miRNA-148a plays a pivotal regulatory function in the liver. On the one hand, it is crucial for hepatic differentiation through the direct targeting of DNA methyltransferase, thereby allowing promotion of the “adult liver” phenotype, and on the other hand, it acts as a tumor suppressor in liver neoplasia [36].

MiRNA-148a was found to be downregulated in various types of cancer, including gastric, colorectal, pancreatic, lung, liver, and breast [37]. Moreover, the expression of miRNA-148a has been linked to the prognosis of several tumors. Patients with reduced expression of miRNA-148a have reduced survival and a more aggressive tumor phenotype [38]. Tumor cell proliferation is inhibited by the upregulation of miRNA-148a, and miRNA-148a is one of the milk-derived exosomal miRNAs that can potentially regulate cell proliferation. To elucidate the question of whether cell proliferation is directly regulated by milk-derived exosomal miRNAs, we used cells expressing stable shRNA that downregulate miRNA-148a. Silencing of miRNA-148a-3p induced the proliferation of HEK-293T cells, and incubation of silenced miRNA-148a HEK-293T cells with MDEs inhibited their growth. This finding confirms that the effect of MDEs on cell proliferation is mediated by their miRNA cargo. In addition, miRNA-148a silencing shows for the first time that the effect of exosomes on gene expression is dependent on this cargo. We demonstrate that the effect of MDEs on DNMT1 and PTEN protein expression is milk-miRNA dependent.

In our current study, one of our main finding was the bimodal action of MDE in a linage dependent manner. There may be several options to explain the dual effect of MDE on tumor and normal cells. Based on the results of this study we hypothesize that regulation by miRNA such as miRNA148a, can be one of the mechanisms. For example, miRNA-148a have a dual effect on different tumor cells. MiRNA-148a has on one hand a tumor suppressor activity on hepatecular carcinoma [39], non-small cell lungs carcinoma [40],esophageal cancer [41] by inhibiting cell proliferation among other functions. On the other hand, miRNA-148 promotes cell growth and inhibits cell death in glioblastoma [42].We demonstrated that DNMT1, one of the key enzymes of DNA methylation, is regulated in a miRNA dependent manner by miRNA-148a. Methylation function is to silence gene expression. By regulation of methylation via DNMT1-miRNA-148a pathway, MDE can inhibit different gene that are relatod to tumor or normal cells. Based on these results further studies are needed to explore the mechanism involved in regulation of cell functions by MDE.

## Conclusions

In conclusion, the biological effect of exosomes on cells is miRNA-dependent. MDEs induced proliferation and protein expression in a miRNA-dependent manner. Furthermore, MDEs induced different biological effects on normal compared to colon tumor cells. In normal cells, MDEs have a beneficial effect by inducing EMT and cell proliferation, which are crucial for intestinal growth and development. In contrast, these positive effects were not observed in tumor cells. MDEs promote normal epithelial but not tumor cell proliferation and protein expression in a miRNA-dependent manner. This suggests that exosome administration may be a valuable nutritional supplement for infants fed with formula in helping their normal development.

## Supplementary information

**Additional file 1.** Morphology of colonic normal cells incubated with MDE isolated from cow milk.

**Additional file 2.** Morphology of colonic tumor cells incubated with MDEs.

## Data Availability

The datasets used and/or analyzed during the current study are available from the corresponding author on reasonable request.

## Abbreviations

MDE: milk-derived exosome
DNMT1: DNA methyltransferase 1
EMT: epithelial–mesenchymal transition
miRNA: microRNA
PTEN: phosphatase and tensin homolog
