# Supplementary material for: Milk-derived exosomes (MDEs) have a different biological effect on normal fetal colon epithelial cells compared to colon tumor cells in a miRNA-dependent manner
Source: J Transl Med. 2019 Sep 30;17:325. doi: 10.1186/s12967-019-2072-3 (PMC6767636; doi:10.1186/s12967-019-2072-3)
Supplement: Supplementary file 1 — Additional file 1. Morphology of colonic normal cells incubated with MDE isolated from cow milk. [file 12967_2019_2072_MOESM1_ESM.pdf]

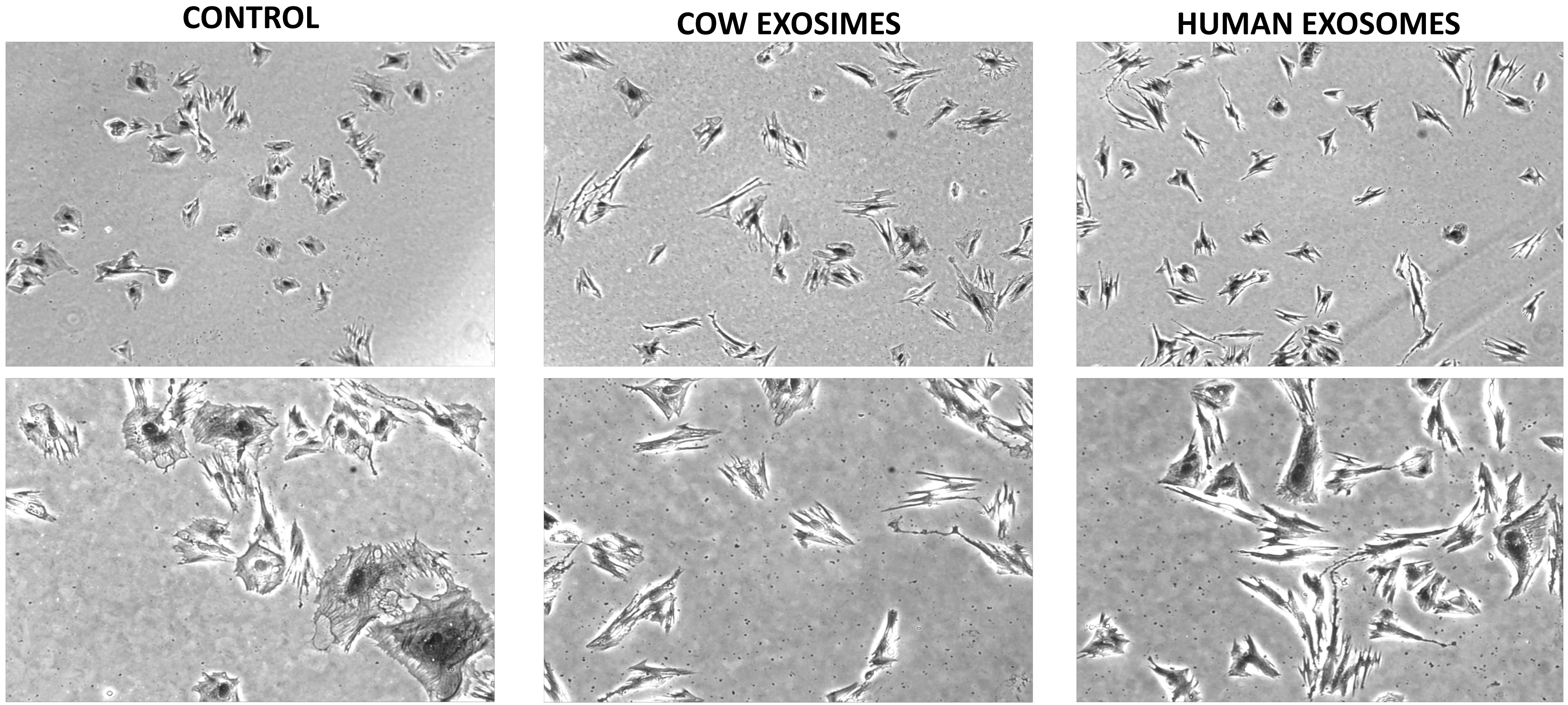

Figure S1. Morphology of colonic normal cells incubated with MDE isolated from cow milk. Exosomes were grown in 0% FCS and incubated with colonic normal cells (CCD 841). Light microscope pictures of selected fields of cells incubated with cow and human exosomes (COW EXOSOMES) , (HUMAN EXOSOMES) or without (CONTROL). MDE were isolated by sequential centrifugations. Briefly, following milk fractionation the skim was filter by 0.45μM and 0.22μM filters. The filtered skim was centrifugated at at 100000 g for 1 hour at 4°C to remove casein. To isolate MDE the supernatant was centrifugated at 135000 g for 1.5 hour at 4°C and the pellet are the MDE.
