# Supplementary material for: Milk-derived exosomes (MDEs) have a different biological effect on normal fetal colon epithelial cells compared to colon tumor cells in a miRNA-dependent manner
Source: J Transl Med. 2019 Sep 30;17:325. doi: 10.1186/s12967-019-2072-3 (PMC6767636; doi:10.1186/s12967-019-2072-3)
Supplement: Supplementary file 2 — Additional file 2. Morphology of colonic tumor cells incubated with MDEs. [file 12967_2019_2072_MOESM2_ESM.pdf]

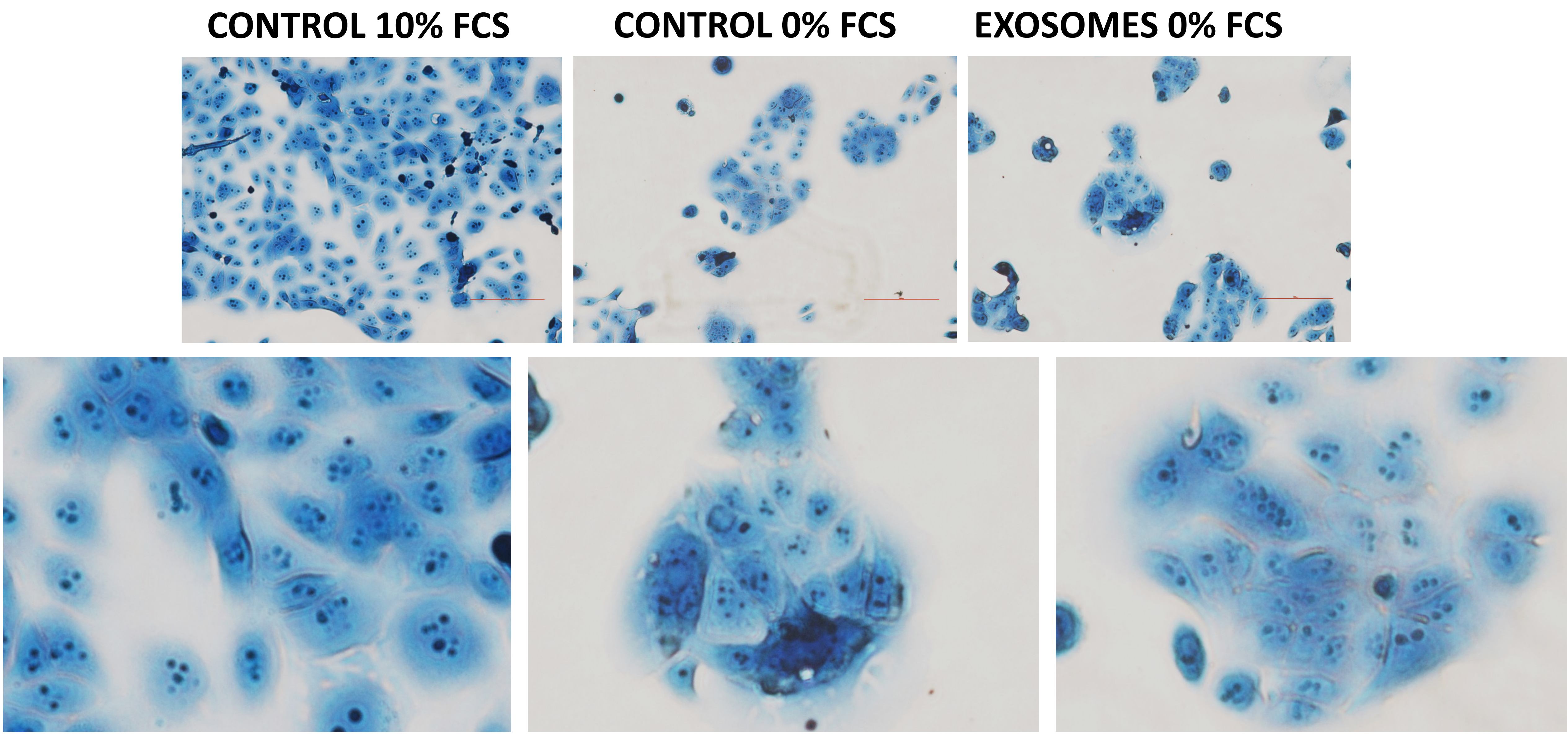

Figure S2. Morphology of colonic tumor cells incubated with MDEs . MDEs were grown in 10% or 0% FCS and incubated with colonic tumor cells (LS123). Light microscope pictures of selected fields of LS123 cells incubated with MDEs (EXOSOMES) or without (CONTROL) following methylene blue staining.
